# Orthopaedic Eponyms: A Tool of the Past

**DOI:** 10.7759/cureus.43336

**Published:** 2023-08-11

**Authors:** Edward Perera, Akib Khan, Khaled M Sarraf, Dominic Spicer

**Affiliations:** 1 Trauma and Orthopaedics, Imperial College Healthcare, London, GBR; 2 Trauma and Orthopaedics, Northwick Park Hospital, London, GBR

**Keywords:** surgery, history of medical sciences, medical humanities, medical education and training, eponyms, orthopaedics surgery

## Abstract

Eponyms are commonplace in the medical vernacular, however, their use has become increasingly controversial amongst clinicians. Whilst some view them as an honour bestowed on those whose achievements deserve recognition, others see them as thwarted with problems due to confusion, imprecision and unwittingly applauding controversial figures. Nevertheless, the history and culture retained within eponyms define modern-day medicine. To identify current trends in understanding of eponyms, we presented a questionnaire of orthopaedic eponyms and their associated imaging to unspecialised trainees, specialist orthopaedic trainees, and qualified consultants. Eponymous terms were poorly understood at all levels of experience, with- third and fourth-year Orthopaedic trainees (specialist trainee years five and six (ST5/ST6)) being outperformed (22.3%) by non-specialist postgraduate doctors with two or more years of experience (foundation year two (F2) and core surgery year two (CT2)) (29.3%). Based on these trends we present a further narrative review of the challenges eponyms present, whilst justifying their continued use to acknowledge the origins of our discipline, from the favourable to shameful.

## Introduction

Eponyms are commonplace throughout medicine. Their roots lie in the historical figures after whom they are named, and to whom much of modern medicine owes tribute. One such example is the term Charcot foot or joint, described by Jean-Martin Charcot for neuropathic osteoarthropathy. The disease refers to the peripheral loss of sensation and proprioception resulting in the absence of protective feedback from repetitive microtrauma. This leads to damage to skin integrity, soft tissue and bone infection, bony destruction and deformity of the joint, and eventual loss of function or loss of limb [1]. The role of the eponym is to concisely convey detailed information, which would otherwise require a lengthy description that may overcomplicate or confuse [2].

Clinicians are often required to make quick and accurate decisions in time-pressured environments. It would follow that improved efficiency in conveying information through the use of eponyms would be reasonable and perhaps invaluable. Descriptive methods can be cumbersome to communicate, which also may favour the use of eponyms, whose understanding conveys specific information relating to the condition, its causes, and its associations.

However, this is only the case if eponyms are accurately understood. Arguments against the use of eponyms are that they are an outdated and archaic form of communication. Learning the vocabulary of medicine is like studying another language, there are already a multitude of medical conditions to learn and an ever-increasing number of classification systems [3]. The necessity for further knowledge of eponymous terms can often confuse clinicians, and the attached understanding of conditions differs between clinicians [4]. For example, in ankle fractures alone there exist greater than nine different eponymous terms, which potentially only aids to confuse rather than clarify [2, 5].

Others would argue that many of the eponymous terms we use must be updated either due to inaccuracies of the original describer, who some have found may not have even been the first to identify the traits or pathology they describe [6]. Another concern is due to historical events, such as those 20th-century physicians with sympathies towards totalitarian regimes or ethically unjustifiable practices. Hugo Spatz and Julius Hallervorden, attributed with Hallervorden-Spatz syndrome, a neurological disorder characterised by dystonia, parkinsonism, and iron accumulation in the brain, conducted research on brain tissue removed from children killed under the euthanasia project in Nazi Germany. Such figures are less than desirable to be immortalised in our current practice [7-9]. However, obscuring such terms from our vocabulary may be construed as negationism and work to gloss over some of the unsightly origins of modern medicine. The education of new clinicians as to the origins of their medical routes lies in understanding the impact of our predecessors in shaping our modern medical culture.

Medicine is a culture of international contribution and collaboration, and this is directly visible through our eponyms. In 1912, Hakaru Hashimoto of Japan first identified what he termed struma lymphomatosa, at the time a new form of lymphoid and plasma cell infiltration within the thyroid. At the time, Hashimoto was training in Germany, however, his findings were seen to be indistinct from Riedel’s thyroiditis, a form of chronic thyroid inflammation. It was not until 1931 when an American, Allan Graham, supported his assertions that the term Hashimoto’s thyroiditis was coined. Eponyms not only demonstrate global contributions to the field but are used in all corners of the globe [10].

We hypothesise that level of understanding of eponymous terms among orthopaedic clinicians is generally poor, but that knowledge will peak as trainees approach consultancy and then decline as they become more specialised. We will further provide a narrative review of the current challenges with the use of eponymous terms and will argue that this should be an area for possible development and revival to improve clinical practice and efficiency, as well as the overall appreciation of our medical culture.

This article was previously presented at the 2022 42nd SICOT Orthopaedic World Congress on 28-30 September, 2022 and the 2022 British Trauma Society Annual Scientific Meeting on23-24 November, 2022.

## Materials and methods

Two authors (EP and AK) produced an orthopaedic questionnaire focussing on the correct matching of eponymous fractures and orthopaedic pathology with their respective radiographic imaging, or illustrations of pathology. The included topics were chosen via consensus decision to appropriately cover a diverse range of levels of knowledge, which would be expected of trainees at different stages in their training.

We presented the questionnaires at live events through the medium of a Microsoft PowerPoint presentation (Microsoft Corporation, Redmond, USA) (see Appendices). A total of 25 eponymous conditions over 25 slides were assessed. Each slide consisted of five different radiographic or illustrative options with 15 seconds given per slide to review the images. Answers were gathered anonymously, with participants using a quick response (QR) code linked to a Google Forms answer sheet (Google LLC, Mountain View, USA).

We collected data at separate training events for foundation trainee doctors (non-specialised trainees), senior house officers (post-two years of foundation training but non-specialised in orthopaedics), senior trainees (specialist trainees in orthopaedics), and consultant orthopaedic surgeons within the London region.

We used descriptive statistics (mean, median, range, standard deviation (SD), and percentages as appropriate) to summarise collected data and performed analysis with Microsoft Excel (version 16.66.1, Microsoft Corporation, Redmond, USA) by one author (EP) with the consultation of senior authors (AK, KS, and DS).

Informed consent was obtained from all individual participants included in the study. Ethical approval was not deemed necessary by the local committee as the research involved the use of non-sensitive, completely anonymous educational tests and surveys where participants were not defined as “vulnerable” and participation would not induce any undue psychological stress or anxiety.

## Results

A total of 34 clinicians completed the questionnaire. Of these, 12 were considered junior trainees (foundation trainees and senior house officers) and 22 were specialists (specialist orthopaedic trainees or qualified orthopaedic consultants). A total of 25 different eponymous terms were assessed, these were subdivided into the following sections: seven hand, wrist, and forearm; two upper arm and shoulder; four spine, hip and pelvis; three knee; and nine ankle. Amongst junior trainees, the most correctly identified eponymous term was the Chauffeur’s fracture (54.5%), and amongst specialist trainees, the most correctly identified orthopaedic eponymous terms were Lisfranc, Segond, and Pilon fractures (95.5%) (Table 1).

**Table 1:** All eponymous orthopaedic terms questioned in the questionnaire, percentage correct, and the most commonly chosen answer

Eponymous term	Description	% Correct within Cohort		Most commonly chosen answer (%)	
		Junior trainees (non-specialist trainee)	Senior trainees (Orthopaedic specialist trainee)	Junior trainees (non-specialist trainee)	Senior trainees (Orthopaedic specialist trainee)
Smith’s Fracture	A distal radius fracture with volar displacement and/or angulation of the distal fragment.	18.2%	86.4%	Colles (36.4%)	Smith’s (86.4%)
Holstein-Lewis Fracture	A distal 1/3^rd^ spiral fracture of the humeral shaft. with a radially deviated proximal end increasing the risk of acute radial nerve palsy.	30%	45.5%	Supracondylar (40%)	Mid-shaft humeral fracture with ulnar displacement / Holstein-Lewis (45.5%)
Monteggia	Proximal 1/3^rd^ ulnar shaft fracture w/ radial head dislocation.	30%	90.5%	Chauffeur / Monteggia / Galeazzi (30%)	Monteggia (90.5%)
Jones	Extraarticular 5^th^ metatarsal proximal diaphyseal fracture, distal to the tuberosity.	9.1%	81%	5^th^ Metatarsal spiral shaft fracture (45.5%)	Jones (81%)
Chauffeur	A distal radius fracture with volar angulation of the distal fragment.	54.5%	86.4%	Chauffeur (54.5%)	Chauffeur (86.4%)
Aviator’s Astragalus	Forced dorsiflexion of the ankle, producing a coronal-plane fracture of the neck of the talus.	9.1%	22.7%	Chopart (36.4%)	Subtalar dislocation (59.1%)
Galeazzi	Fracture of the middle to distal 1/3^rd^ of the radius, with associated distal radio-ulnar joint (DRUJ) injury / dislocation.	36.4%	90.9%	Galeazzi (36.4%)	Galeazzi (90.9%)
Lisfranc	A traumatic dislocation between the articulation of the medial cuneiform and base of the second metatarsal.	50%	95.5%	Lisfranc (50%)	Lisfranc (95.5%)
Chopart	A fracture dislocation of the foot through the talonavicular and the calcaneocuboid joints.	0%	4.5%	Subtalar dislocation (44.4%)	Tillaux / Charcot Joint (31.8%)
Wagstaffe-le fort	An avulsion to the medial aspect of the distal fibula due to avulsion of the anterior tibiofibular ligament (ATFL) attachment.	11.1%	22.7%	Aviators Astragalus (44.4%)	Tillaux-Chaput (63.6%)
Rolando	A three-part intra-articular fracture with dislocation of the base of the 1^st^ metacarpal.	10%	63.6%	Boxer’s (40%)	Rolando (63.6%)
Kohler	Self-limiting, avascular necrosis (osteonecrosis) of the navicular bone in children.	33.3%	50%	Kohler (33.3%)	Kohler’s (50%)
Schatzker type II	Lateral tibial plateau fracture with a split and depressed lateral component.	30%	54.4%	Schatzker Type II (30%)	Schatzker Type II (54.5%)
Bosworth	Fracture of the distal fibula with an associated fixed posterior dislocation of the proximal fibular fragment, behind the posterior tibial tubercle.	25%	36.4%	Tillaux / Weber B / Bosworth (25%)	Subtalar dislocation (40.9%)
Bennett	An intraarticular fracture of the base of the 1^st^ metacarpal of the thumb.	33.3%	27.3%	Bennett / Rolando (33.3%)	Rolando (63.6%)
Tillaux	Salter-Harris III fractures through the anterolateral aspect of the distal tibial epiphysis, with variable amounts of displacement.	20%	59.1%	Subtalar dislocation (40%)	Tillaux (59.1%)
Segond	Avulsion fracture of the lateral aspect of the tibial plateau, associated with anterior cruciate ligament (ACL) tear.	12.5%	95.5%	Schatzker Type I (50%)	Segond (95.5%)
Garden Type III	Complete neck of femur (NOF) fracture with varus position and partially displacement.	18.2%	36.4%	Garden Type IV (54.5%)	Garden Type IV (50%)
Gartland Type IIa	Suprachondylar fracture with intact posterior cortex with posterior angulation. The anterior humeral line and capitellum, do not intersect.	11.1%	72.7%	Gartland Type Ib (66.7%)	Gartland Type IIa (72.7%)
Vancouver Type B3	Fractures of the femoral diaphysis at the level of the stem or just below it with evidence of stem loosening and severe bone stock loss.	0%	50%	Vancouver B2 / Vancouver A (30%)	Vancouver Type B3 (50%)
Duverney	Fracture to the iliac wing due to lateral compression of the iliac wing, often secondary to a direct blow	11.1%	4.5%	Acetabular fracture (44.4%)	Acetabular fracture (63.6%)
Burst	Compression fracture through axial loading of the spine, causing retropulsion of posterior vertebral body cortex into the spinal canal.	45.5%	86.4%	Burst (45.5%)	Burst (86.4%)
Hoffa	Fracture of the distal femoral condyle associated with a coronal plane fracture.	33.3%	86.4%	Hoffa (33.3%)	Hoffa (86.4%)
Pilon	An intraarticular fracture of the distal tibia, associated with comminution.	20%	95.5%	Hawkins Type I (30%)	Pilon (95.5%)
Colles	A distal radius fracture with dorsal displacement and/or angulation of the distal fragment.	27.3%	90.9%	Smith’s (36.4%)	Colles (90.9%)

**Figure 1:**
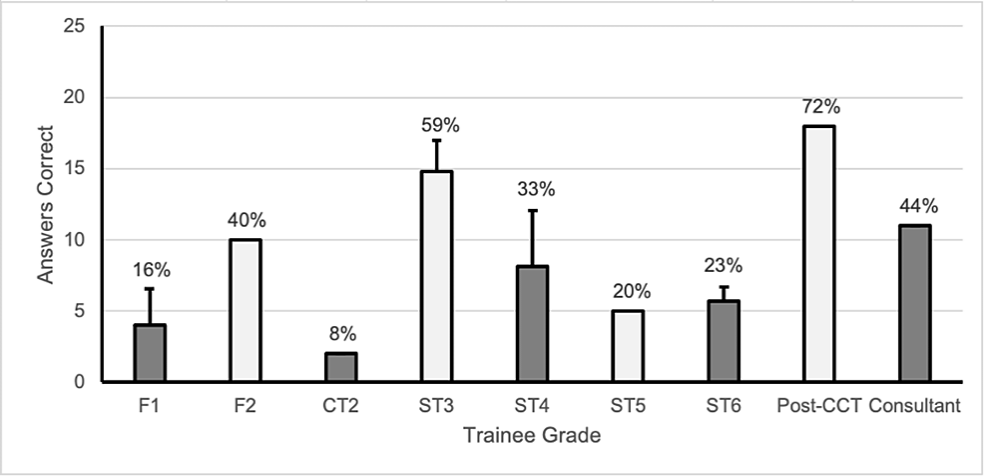
The mean number of answers correct according to training grades The equivalent percentage correct according to training grade is included as a figure above the bars. Whiskers above the bars indicate standard deviations (SD); where no whisker exists, the SD was not calculable. F1-2 = Foundation Year 1-2; ST/CT2 = Specialty/Core Training Year 2; ST3-6 = Specialty Training Year 3-6; Post-CCT: Post-Certificate of Completion of Training

Clinicians were subdivided within their junior or specialist groups into years of training. One surgeon in the specialist group was a post-certificate of completion of training fellow (CCT) and was categorised as a consultant for purposes of subgrouping. Amongst junior trainees, the mean number of correct answers for the questionnaire was four (16%) with a standard deviation (SD) of 1.71 and a range from one (4%) to seven (28%) correct answers. Amongst specialist trainees and qualified orthopaedic surgeons, the mean number of correct answers for the questionnaire was 15.3 (61%) with an SD of 3.18 and a range from nine (36%) to 21 (84%) correct answers. As a group, foundation year one trainees performed the worst and ST3 trainees performed the best in the questionnaires (Table 2). The lowest-performing individual was a foundation year one doctor and the highest-performing individual was a CCT Fellow (Figure 1).

**Table 2:** Participant characteristics and mean values for correct answers F1 = Foundation Year 1; SHO = Senior House Officer; ST3-5 = Specialty Training Year 3-5.

	Level	Total number	Mean correct answers (SD)	Mean correct answers as a percentage
Junior Trainees	F1	9	4 (2.55)	16%
	SHO	3	7.33 (4.62)	29.3%
Specialist Trainees & Consultants	ST3	5	14.8 (2.17)	59.2%
	ST4	8	8.13 (3.94)	32.5%
	>ST5	7	5.57 (0.98)	22.3%
	Post-qualification	2	14.5 (4.95)	58%

We found that the level of knowledge of orthopaedic eponymous terms did not follow a linear progression with respect to the level of seniority. We noted that first-year specialist (ST3) trainees performed the best as a group, with a gradual decline in knowledge of orthopaedic eponyms until post-qualification. Across both groups, the knowledge of hand, wrist, and forearm, and knee eponyms was the greatest; whilst juniors performed worst in foot and ankle eponyms and specialists performed worst in spine, hip, and pelvis eponyms (Table 3).

**Table 3:** The mean and percentage of correct response based on the anatomical region of eponymous terms N indicates the number of participants per group.

		Hand, Wrist & Forearm	Upper Arm & Shoulder	Spine, Hips & Pelvis	Knees	Foot & Ankles
Junior Trainees (n=12)	Mean correct response	3.14	2	2	2.33	1.89
	Percentage correct	26%	17%	17%	19%	16%
Specialist trainees & Consultants (n=22)	Mean correct response	16.7	13	9.75	17.3	11.3
	Percentage correct	76%	59%	44%	79%	52%

## Discussion

The primary finding of our study was that substantial confusion regarding eponymous terms exists at all levels of seniority, in keeping with our stated hypothesis. In addition, we interestingly noted that whilst knowledge of orthopaedic terms increased with level of training and seniority, this had a bimodal distribution, peaking in the first year of specialist training and again post-qualification. This finding was not in keeping with our hypothesis, that as specialist trainees approached their consultancy examination their knowledge would peak and this would the fall after.

As we had expected, the understanding of eponymous orthopaedic terms creates confusion amongst clinicians, regardless of level of training or seniority. One reason for this factor may be the sheer multitude of terms that exist. One such example was in the understanding of eponymous foot and ankle terms. We included a total of nine eponymous injuries relating to the foot and ankle, the most of any anatomical region assessed. This was the worst-performing region amongst juniors and the second worst among specialists.

From Figure 1 we see that first-year specialist trainees (ST3s) had the best understanding of eponymous terms, however trainees with more experience in the specialty demonstrated poorer knowledge. However, we observe a second peak at the level of the post-CCT fellow. When we couple this with the previous peak around the entry into specialty training, it is possible that the presence of exams may influence this distribution. Entry into specialty training, whilst interview based, follows a clinical examination and viva format, similar to that of the Royal College examinations. Whilst eponymous terms may not be directly tested within interviews and examinations, one might argue that knowledge and understanding of these terms are integral to success at these two transition points. It is possibly this fact that accounts for the bimodal distribution in the knowledge of eponyms.

Somewhat surprisingly, a specialist trainee greater than five years after completing foundation training (>ST5) performs on average worse than a senior house officer (SHO), who has not secured an orthopaedic training post. It is possible that the focus of training on an academic basis for management means that trainees favour more scientific and evidence-based systems. From the distribution we observe, we might imply that as clinicians develop and mature within the specialty, they come to see eponyms as outdated and of little clinical use and begin to disregard the importance of eponymous terminology. However, preparation for examinations may lead trainees to discover or rediscover these eponymous terms. Whilst a second peak at the post-CCT fellow may suggest that as trainees look to organise the knowledge they reencounter these eponyms, we postulate that these orthopaedic eponyms may also represent a valuable learning tool in those much more senior approaching consultancy. We would argue that where trainees are more focused on scientific systems they may be missing out on the value and knowledge offered in eponyms, that may only be realised towards the end of their training.

One such solution to this challenge is a universal classification to allow consistent understanding, which we see in the Müller Arbeitsgemeinschaft für Osteosynthesefragen (AO) classification of fractures first published in 1987 [11]. This provides a system of numbers and letter to classify the bone, area affected, whether this involves the articular surface and the complexity of the fracture itself. However, pure descriptive terms may be just as confusing as eponymous terms, although this is not something we assessed within our questionnaire. As Wright points out the use of Crohn’s disease is widely used, however, the descriptive term, known to be regional ileitis is a misnomer, since the condition is not confined to the ileum [12].

As for the AO classification system, it has its own drawbacks. A 23-A2 (a distal radius, extra-articular, simple, impacted fracture) does not communicate the mechanism or deforming forces that lead to the fracture. In comparison the eponymous Colles' and Smith’s fracture inform us that the distal fragment is either dorsally or volarly angulated, and likely result from falling forward or backward onto an outstretched hand, respectively. The other argument against this form of classification as a rather cold and unemotional way in which the fracture is described, by simple numbers and letters and that “eponyms bring colour to medicine” [13].

Nonetheless, this vibrancy and history that eponyms bring to our practice becomes redundant if it impedes or is counterproductive to providing concise and accurate communication of information between clinicians. Whilst our study suggested that eponyms of the hand, wrist, and forearms were better understood, whilst 86.4% and 90.9% of specialist correctly identified the Smith’s and Colles' fracture, the remaining mistook it for the other, which was also the case with the Monteggia (90.5%) and Galeazzi (90.9%) fractures. This is likely due to their relative opposition in terms of description with respect to dorsal versus volar angulation (Colles' versus Smith’s) and dislocation of radial head versus distal ulna (Monteggia versus Galeazzi). This is in agreement with the work of Waseem et al. who found that only 10.7% of clinicians were correctly performing the Finklestein’s test, even though 90.4% of those assessed stated they regularly used it [4]. However, it should be noted that a potentially confounding factors exists in individuals’ interpretation of radiographic images. It is possible that some of the more junior trainees may have been aware of the information conveyed by the eponymous term, however, due to inexperience in interpretation of radiographs they selected the relative opposing condition, such as confusing a Colles' and Smith’s.

Another common criticism of eponymous terms is the association of terms with less than savoury historical figures, whose contributions to medicine would preferably not be applauded. In the rebranding of eponymous conditions, often Nazi supporters and sympathisers are used as examples [14]. The well-known condition previously known as Wegener’s Granulomatosis became Granulomatosis with Polyangiitis, due to Friederich Wegener’s experimentation on the mostly Jewish and Romany detainees of Lodz Concentration Camp in Poland. He also remained silent about the events and genocide that took place within and around the camp in the trials of Nazi War Criminals at the end of World War II [15]. In 2000 Wegener’s Nazi links came to light, which resulted in the American College of Chest Physicians revoking a previously awarded Master Clinician award in 2007, after these links were highlighted to them [16]. The story behind eponyms can sometimes help in recollection of the pathology. For example, many a clinician may recall being asked: “and why do we not call it Wegener’s Granulomatosis anymore?”, to which end many clinicians know of his Nazi and criminal past, an argument made by Liao et al. in incorporating medical humanities into current teaching [17]. Some have suggested that the finding of such eponyms should initiate their replacement with eponyms to honour those who opposed or protested such individuals [8].

Whilst during our research we could not identify any specific orthopaedic eponyms with disreputable namesakes, it is possible that in the study of anatomy, orthopaedic surgeons may come across *Pernkopf’s Atlas of Topographical and Applied Human Anatomy*. Pernkopf was a supporter of the Nazi party, being principally involved in the University of Vienna’s removal of Jewish staff and students. Pernkopf’s illustrators were seen to have incorporated Nazi symbols in their signatures to their illustrations and there has been postulation that victims of Nazi atrocities may have made their way into the illustrations of the *Atlas*, although evidence remains unclear [18]. The ethical considerations over the continued use of such texts are complex [19], however, we should not forget the name Pernkopf, in part for his own transgression, but equally to remember the victims closely connected with him and such oppressive regimes. It is essential that those succeeding the specialty continue to question and ethical critique our predecessors and we do not completely obliterate such stains from our past to the point where we cease to recall them.

Despite the aforementioned issues with eponymous names that have led some to question the longevity of their usage, we see the potential for their continued use [13]. However, as a community we should either decide to embrace them and teach them properly to reduce errors, or whether they should be confined to the history books as we move on to more standardised versions of description.

Limitations

Whilst our study has demonstrated generally poor level of understanding of orthopaedic eponyms amongst both specialists and non-specialised trainees, there is a lack of power of conclusions due to the limited sample size. Furthermore, conclusions drawn relating to overall group and subgroup performance may not be generalisable due to the inherent sampling bias occurring from selection of participants based on geographical location.

## Conclusions

Eponymous terminology is in general poorly understood by both junior non-specialised trainees and senior orthopaedic trainees, with a few exceptions. Senior trainees demonstrated some of the worst knowledge, which should be a marker of concern for educators and trainers. The history and culture retained within these eponymous names define much of modern-day medicine and we should ensure our training is as focused on where we have come from as on where we are going. Whether to acknowledge great contributions or to not forget those whose ethics and morality harmed innocents and shamed the profession, we believe eponyms still have their place in our practice.

## Figures and Tables

**Figure 2:**
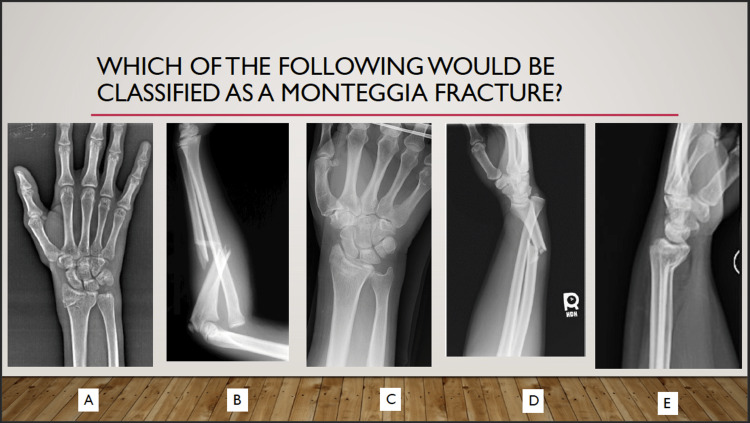
Sample of Microsoft PowerPoint slide from the questionnaire presented to participants.
